# Une histoire de fesse: cas d'une tuberculose gommeuse de la fesse chez un adulte immunocompétent

**DOI:** 10.11604/pamj.2020.37.390.26223

**Published:** 2020-12-31

**Authors:** Aminata Deh, Boubacar Ahy Diatta, Saer Diadie, Abdou Madjib Gaye, Khadim Diop, Niar Ndour, Maodo Ndiaye, Moussa Diallo, Oumou Suzanne Niang

**Affiliations:** 1 Service de Dermatologie, Hôpital Aristide Le Dantec, Dakar, Sénégal,; 2 Service d´Anatomopathologie, Hôpital Aristide Le Dantec, Dakar, Sénégal

**Keywords:** Tuberculose gommeuse, fesses, Sénégal, Pulmonary tuberculosis, buttocks, Senegal

## Abstract

La tuberculose est une maladie endémique au Sénégal. Les formes cutanées sont rares et se caractérisent par leur polymorphisme clinique. Elles représentent 2% des localisations extra pulmonaires de la tuberculose. Nous rapportons l'observation d'une gomme tuberculeuse de la fesse révélant une localisation pulmonaire active chez un adulte immunocompétent. Un homme âgé de 47 ans fut admis pour une tuméfaction douloureuse de la fesse droite évoluant depuis quatre ans. L'examen physique notait un placard induré et polyfistulisé fait de nodules confluents avec une émission de pus jaunâtre, siégeant à la face inférointerne de la fesse droite, associé à des adénopathies inguinales bilatérales d'allure inflammatoire. Une tuberculose cutanée était retenue devant l'histologie qui montrait un granulome tuberculoïde et la recherche de BAAR dans le liquide de tubage gastrique qui était positif. La tomodensitométrie thoraco-abdominopelvienne avait montré de multiples micronodules pulmonaires acinaires basals bilatéraux. Les sérologies VIH et VHB étaient négatives. Une guérison était obtenue six mois après un traitement antituberculeux. La tuberculose cutanée se caractérise par son polymorphisme clinique en zone d'endémie. Elle doit aussi être cherchée devant tout placard d’abcès du périnée.

## Abstract (en)

Tuberculosis is an endemic disease in Senegal. It rarely affects the skin. In these patients it is characterized by clinical polymorphism. It accounts for 2% of extra-pulmonary tuberculosis. We here report the case of an immunocompetent patient with tuberculosis affecting the buttock characterized by lesions gummy in consistency revealing active pulmonary involvement. This study involved a 47-year-old man admitted for a painful swelling in the right gluteal region that had evolved for four years. Physical examination objectified a polyfistulized hardened plaque characterized by confluent nodules and discharge of yellowish pus in the lower and inner face of the right buttock associated with bilateral inguinal inflammatory adenopathies. The diagnosis of skin tuberculosis was retained based on histological examination which showed tuberculoid granuloma and the detection of Alcohol-Acid Resistant Bacilli (BAAR) in the gastric fluid. Thoracoabdominopelvic CT scan showed multiple bilateral basal lung micronodules with a “bud tree” appearance. Serologic testing for HIV and HBV were negative. The patient achieved recovery after six months of tuberculosis treatment. In endemic areas, skin tuberculosis is characterized by clinical polymorphism. It should be suspected in patients with perineal abscess lesion.

## Introduction

La tuberculose est une maladie endémique au Sénégal. Les formes cutanées sont rares et se caractérisent par leur polymorphisme clinique. Elles représentent 2% des localisations extra pulmonaires de la tuberculose [1, 2]. Le diagnostic de la tuberculose cutanée est souvent rendu difficile en raison du faible rendement bactériologique et repose parfois sur des critères présomptifs. Nous rapportons une observation d´une gomme tuberculeuse de la fesse révélant une localisation pulmonaire active chez un adulte immunocompétent.

## Patient et observation

**Figure 1:**
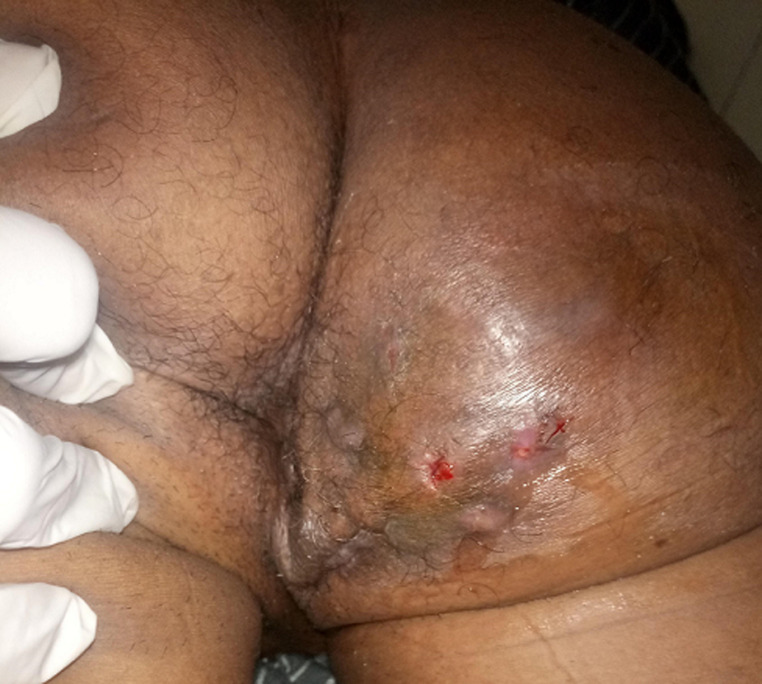
placard multinodulaire, infiltré et polyfistulisé de la fesse droite

Un homme âgé de 47 ans, originaire du Fouta (nord du Sénégal), était hospitalisé pour une tuméfaction douloureuse de la fesse droite évoluant depuis quatre ans. Il décrivait une toux chronique non productive depuis deux mois. Il n´avait pas de fièvre mais présentait un amaigrissement (IMC à 15kg/m^2^). L´examen physique objectivait un placard multinodulaire et polyfistulisé avec une émission de pus jaunâtre, siégeant à la face inférieure et interne de la fesse droite (Figure 1). Par ailleurs, il existait aussi des adénopathies inguinales bilatérales d´allure inflammatoire sans tendance à la fistulisation avec une peau en regard d´aspect normal. L´auscultation et la percussion thoraciques révélaient un syndrome de condensation pulmonaire basal bilatéral. La sérologie rétrovirale était négative, la glycémie à jeun, les fonctions rénale et hépatique étaient normales.

**Figure 2:**
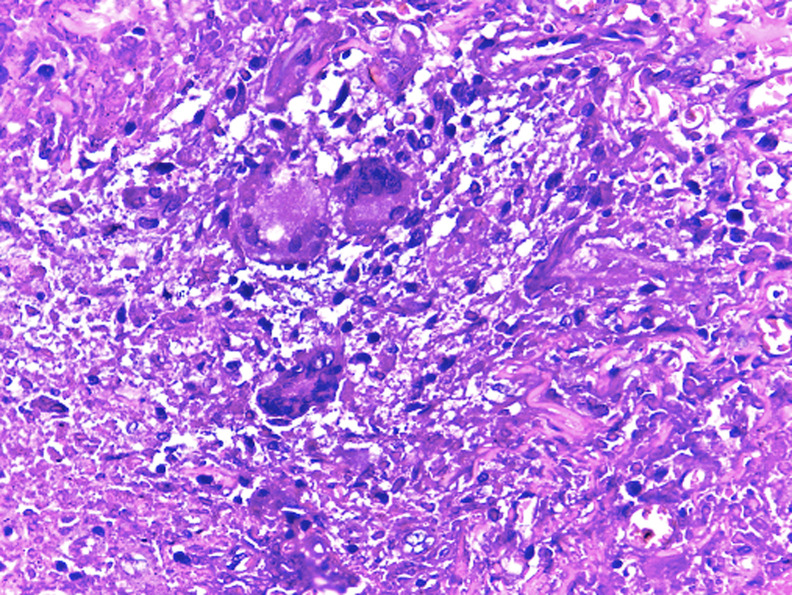
granulome tuberculoïde HE x 400

**Figure 3:**
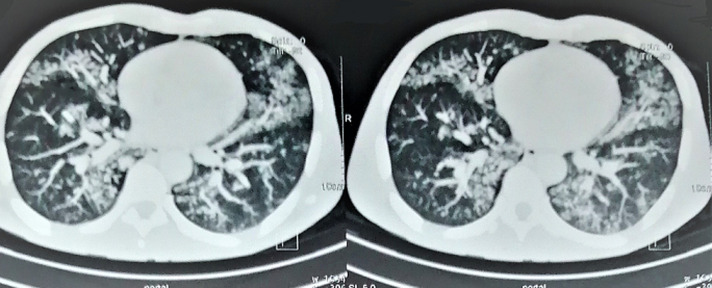
multiples micronodules pulmonaires acinaires basaux bilatéraux avec un aspect en «arbre à bourgeon»

L´examen bactériologique du pus cutané restait négatif. L´étude histologique de la peau (Figure 2) avait mis en évidence la présence d´un granulome tuberculoïde. La recherche de BAAR et le GenExpert positifs dans le liquide de tubage gastrique. La tomodensitométrie thoraco-abdominopelvienne montrait de multiples micronodules pulmonaires des bases bilatéraux avec un aspect en «arbre à bourgeon» (Figure 3). Cependant, la coloscopie totale était normale et sérologies VIH et VHB étaient négatives. Ainsi, nous avons retenu la tuberculose de fesse dans sa forme gommeuse avec une atteinte pulmonaire active. La maladie de Verneuil, l´actinomycose et le mycétome fongique constituaient les principaux diagnostics différentiels. Un traitement antituberculeux a été instauré avec le protocole national (2RHZE/4RH) associant la rifampicine (R), l´isoniazide (H), la pirazinamide (Z) et l´éthambutol (E) pendant six mois. L´évolution était favorable marquée par une prise de poids de 20kg, une guérison et une cicatrisation des lésions cutanées. La bacilloscopie de contrôle était négative et la tomodensitométrie thoracique de contrôle était normale.

## Discussion

La tuberculose est une maladie endémique au Sénégal. Les formes cutanées sont rares et se caractérisent par leur polymorphisme clinique. Elles représentent 2% des localisations extra pulmonaires de la tuberculose [1, 2]. Le diagnostic de la tuberculose cutanée est souvent rendu difficile en raison du faible rendement bactériologique et repose parfois sur des critères présomptifs. La forme clinique est déterminée par la pathogénicité du micro-organisme, la voie d´infection, la sensibilisation préalable du patient à la tuberculose et la nature de l´immunité à médiation cellulaire du patient [3]. Les gommes tuberculeuses sont rares et correspondent à la dissémination hématogène des bacilles à partir d´un foyer tuberculeux profond. Elles représentaient moins de 10% des tuberculoses cutanées dans les études indiennes et anglaises (4,5 et 8%) [4, 5] et moins de 20% dans les études de Niang *et al*. au Sénégal et de Farina *et al*. en Espagne [6, 7].

À notre connaissance, seulement dix cas ont été rapportés dans une étude marocaine sur les causes de suppurations périnéofessières portant sur une période de 21 ans (1987-2008), où la tuberculose cutanée représentait la deuxième cause avec une fréquence de 16,6% [8]. La longue durée d´évolution qui existait chez notre patient est comparable à celle de cette étude où le délai moyen était de 2,3 ans. Les gommes tuberculeuses classées parmi les formes multibacillaires surviennent le plus souvent chez les enfants dénutris ou chez les sujets immunodéprimés. Chez notre patient, nous n´avions pas objectivé un terrain d´immunodépression. Dans l´étude de Gullouj *et al*., chez les 25% qui présentaient une gomme tuberculeuse, il n´y avait pas d´association avec l´infection au VIH [9]. La durée du traitement des gommes tuberculeuses dépend du foyer à l´origine de cette dissémination [10].

## Conclusion

La tuberculose cutanée doit être évoquée devant tout placard cutané fistulisé. La recherche d´un foyer tuberculeux profond est indispensable pour une bonne prise en charge thérapeutique.
